# Development and validation of a prediction model for acute kidney injury following cardiac valve surgery

**DOI:** 10.3389/fmed.2025.1528147

**Published:** 2025-01-31

**Authors:** Xiaotong Jia, Jun Ma, Zeyou Qi, Dongni Zhang, Junwei Gao

**Affiliations:** Department of Anesthesiology, Beijing Anzhen Hospital, Capital Medical University, Beijing, China

**Keywords:** cardiovascular surgery, cardiac valve surgery, acute kidney injury, risk factors, prediction model, external validation

## Abstract

**Background:**

Acute kidney injury (AKI) often accompanies cardiac valve surgery, and worsens patient outcome. The aim of our study is to identify preoperative and intraoperative independent risk factors for AKI in patients undergoing cardiac valve surgery. Using these factors, we developed a risk prediction model for AKI after cardiac valve surgery and conducted external validation.

**Methods:**

Our retrospective study recruited 497 adult patients undergoing cardiac valve surgery as a derivation cohort between February and August 2023. Patient demographics, including medical history and perioperative clinical information, were acquired, and patients were classified into one of two cohorts, AKI and non-AKI, according to the Kidney Disease: Improving Global Outcomes (KDIGO) guidelines. Using binary logistic stepwise regression analysis, we identified independent AKI risk factors after cardiac valve surgery. Lastly, we constructed a nomogram and conducted external validation in a validation cohort comprising 200 patients. The performance of the nomogram was evaluated based on the area under the receiver operating characteristic curve (AUC), calibration curves and decision curve analysis (DCA).

**Results:**

In the derivation cohort, 172 developed AKI (34.6%). Relative to non-AKI patients, the AKI patients exhibited elevated postoperative complication incidences and worse outcome. Based on multivariate analysis, advanced age (OR: 1.855; *p* = 0.011), preoperative hypertension (OR: 1.91; *p* = 0.017), coronary heart disease (OR: 6.773; *p* < 0.001), preoperative albumin (OR: 0.924; *p* = 0.015), D-Dimer (OR: 1.001; *p* = 0.038), plasma creatinine (OR: 1.025; *p* = 0.001), cardiopulmonary bypass (CPB) duration (OR: 1.011; *p* = 0.001), repeat CPB (OR: 6.195; *p* = 0.010), intraoperative red blood cell transfusion (OR: 2.560; *p* < 0.001), urine volume (OR: 0.406 *p* < 0.001) and vasoactive–inotropic score (OR: 1.135; *p* = 0.009) were independent risk factors for AKI. The AUC of the nomogram in the derivation and validation cohorts were 0.814 (95%CI: 0.775–0.854) and 0.798 (95%CI: 0.726–0.871), respectively. Furthermore, the calibration curve revealed that the predicted outcome was in agreement with the actual observations. Finally, the DCA curves showed that the nomogram had a good clinical applicability value.

**Conclusion:**

Several perioperative factors modulate AKI development following cardiac valve surgery, resulting in poor patient prognosis. The proposed AKI predictive model is both sensitive and precise, and can assist in high-risk patient screening in the clinics.

## 1 Introduction

Acute kidney injury (AKI) is a complicated and prevalent condition whereby kidney function decline rapidly over a short period of time. Multiple factors contribute to AKI, for example, insufficient perfusion, ischemia–reperfusion injury, inflammatory responses, oxidative stress, and nephrotoxin exposure (1). Unfortunately, owing to variations in study populations and AKI definitions, the reported Cardiac Surgery-Associated Acute Kidney Injury (CSA-AKI) prevalence is between 3 and 40% (2). Cardiac surgery is the second leading cause of AKI in the intensive care unit (ICU) (3). Valve surgery is an independent risk factor for AKI, and the risk of AKI after cardiac valve surgery is 2.68 times higher than that after coronary artery bypass grafting (CABG) (4). This may be attributed to various factors. Cardiac valve surgery, as it progresses over time, affects the patient’s cardiac function. The presence of preoperative cardiac insufficiency can lead to activation of the renin-angiotensin-aldosterone system (RAAS), the sympathetic nervous system, and vasopressin secretion, resulting in fluid retention (5). Furthermore, cardiac valve surgery necessitates the use of cardiopulmonary bypass (CPB). However, the conditions associated with CPB, such as hemodilution, hypothermia, hypotension, and contact with artificial surfaces, can disrupt kidney microcirculation, activate local and systemic inflammatory responses to non-pulsatile blood flow and low perfusion pressure, thereby increasing the incidence of AKI (6). Additionally, the complexity of the surgery and the greater myocardial injury from cardiac incision underscore the necessity of researching AKI following cardiac valve surgery (7).

CSA-AKI critically modulates patient prognosis, enhances mortality, and prolongs both ICU and hospital length of stay (LOS) (8). According to one meta-analysis, the median pooled short- and long-term mortality rates were 10.7 and 30%, respectively (9). Although the percentage of severe injury patients who require kidney replacement therapy (KRT) is relatively low, i.e., 1–5%, the mortality rate can easily exceed 60% (10, 11). Notably, even minor postoperative kidney injury can considerably reduce patient survival (12, 13), while increasing chronic kidney disease (CKD) and end-stage kidney disease (ESKD) risk in patients (14).

Most researchers are specially focused on developing relevant risk prediction models. Existing scoring models, such as, the Cleveland Clinic (15), Mehta (16), and Simplified Renal Index (SRI) scores (17) predict KRT requirement post cardiac surgery. There are also models for mild, non-dialysis-requiring postoperative AKI, such as the Multicenter Study of Perioperative Ischemia (MCSPI) (18), the Acute Kidney Injury After Cardiac Surgery (AKICS) (19), the Northern New England Cardiovascular Disease Study Group (NNECDSG) scores (20), the CRATE score (21) and the AKI-Pro score (22). Despite the existence of various predictive models for CSA-AKI, most studies have evaluated a broad range of cardiac surgical procedures (23–25). Consequently, specific research focusing on AKI following cardiac valve surgery remains limited. Additionally, existing studies have either only examined preoperative indicators (26, 27) or lacked external validation (28–30). In stark contrast, our study only included patients who received cardiac valve surgery under CPB. At present, a large number of studies have analyzed the risk factors of CSA-AKI, which mainly include adverse clinical status, underlying disease, use of nephrotoxic drugs, contrast injection, some surgical interventions, and anesthetic factors (31). Based on the results of previous studies (32), we not only comprehensively collected preoperative indicators for patients but also assessed intraoperative anesthesia and surgery-associated factors (33). Furthermore, we conducted external validation based on the established predictive model, providing a more comprehensive assessment of risk factors for AKI after cardiac valve surgery. Conclusions from this study will reveal a theoretical foundation for preoperative prevention and intraoperative management.

## 2 Methods

### 2.1 Study design and approvals

Our study is a single-center retrospective observational study in which we developed a predictive model for AKI following cardiac valve surgery and conducted external validation of this model. The derivation cohort comprises patients who underwent elective cardiac valve surgery at Beijing Anzhen Hospital, Capital Medical University, from February to August 2023. The validation cohort includes patients who underwent elective cardiac valve surgery at the same hospital during the period from March to June 2024. The following patients were included in analysis: (1) patients between 18 and 80 years of age; and (2) first-time patients who received cardiac valve surgery with CPB. Patients who were eliminated from analysis: those with (1) pre-existing kidney dysfunction and require KRT; (2) missing clinical information in the institutional medical records; and (3) preoperative complication involving serious injury to important organs (such as cerebrovascular accident, severe lung infection, liver insufficiency and so on).

The study was conducted in accordance with the Declaration of Helsinki (as revised in 2013). The study received ethical approval from the Beijing Anzhen Hospital, Capital Medical University (approval No. KS2023092). Due to the retrospective nature of this study, informed consent was waived.

### 2.2 Clinical data acquisition

For the purpose of clinical research, after obtaining ethical approval and authorization from the hospital’s Information Technology Department, specialists from the Information Department accessed the electronic medical records of participants and collected the following data. During the data collection process, information that could potentially identify individual participants might be obtained. However, the information specialists collecting the data replaced personal information with serial numbers and did not participate in subsequent data processing and statistical analysis. Therefore, it can be ensured that the subsequent analysis was conducted anonymously. (1) preoperative clinical data, such as age, gender, body mass index (BMI), prior underlying diseases, angiography, preoperative laboratory information, such as complete blood count (including white blood cells (WBC), platelets (PLT) and albumin (Alb), etc.), kidney function, coagulation function, left ventricular ejection fraction (LVEF), use of diuretics, angiotensin-converting enzyme inhibitors (ACEIs)/angiotensin receptor blockers (ARBs); (2) intraoperative clinical data, such as intraoperative blood transfusion, bleeding volume, urine volume, nadir hematocrit (HCT), use of vasopressors, fluid balance, surgery category, CPB duration and aortic cross-clamping (ACx) duration; and (3) postoperative clinical information, such as postoperative complications, in-hospital mortality, ICU and hospital LOS.

### 2.3 Study endpoints and definition

AKI diagnosis was defined according to the 2012 Kidney Disease: Improving Global Outcomes (KDIGO) Clinical Practice Guideline for Acute Kidney Injury guidelines (34). The definition of AKI following surgery was an increase in serum creatinine (Scr) by more than 26.5 μmol/L (0.3 mg/dL) within 48 h or more than 1.5 times the baseline level within 7 days. AKI was also staged for severity according to the following criteria: Stage 1: Increase in SCr ≥ 0.3 mg/dL (≥26.5 mmol/L) or 1.5 to 1.9 times baseline; Stage 2: 2.0 to 2.9 times baseline SCr; Stage 3: 3.0 or more times baseline; increase in SCr ≥ 4.0 mg/dL; or initiation of kidney replacement therapy.

The dosage of vasoactive medications administered intraoperatively is expressed using the vasoactive–inotropic score (VIS). VIS = dopamine (μg/kg/min) + dobutamine (μg/kg/min) + 100 × epinephrine (μg/kg/min) + 100 × norepinephrine (μg/kg/min) + 10 × milrinone (μg/kg/min) + 10,000 × vasopressin (units/kg/min) + 50 × levosimendan (μg/kg/min).

### 2.4 Statistical analysis

Continuous variable normality was examined via the Kolmogorov–Smirnov test. Normally distributed continuous data are presented as mean ± standard deviation (±*s*), with inter-group comparisons assessed via the independent samples *t*-test. Data with non-normal distribution are provided as medians with interquartile ranges (*IQR*), with inter-group comparisons made with the nonparametric Mann–Whitney *U* Test or Kruskal-Wallis tests. Categorical information are provided as frequencies and percentages, and assessed using the *χ2* test (and with fisher’s exact probability method when conditions were not met).

Variables achieving *p* < 0.05 in univariate analysis were entered into multivariate analysis. Binary logistic stepwise regression, with confounder adjustment for gender and BMI, was used to evaluate the independent risk factors for post-cardiac valve surgery AKI. Variance inflation factors (VIFs) was calculated to assess the collinearity assumption, with VIF less than 5 considered to indicate no significant collinearity. Lastly, a predictive model was generated, and to facilitate its clinical use, a nomogram was drawn based on the weight of each variable in the model. Further, the nomogram was used to predict postoperative AKI within the derivation cohort.

The predictive performance of the model was subsequently evaluated in patients from the validation cohort. The discrimination of the nomogram was assessed by calculating the area under the receiver operating characteristic (ROC) curve. The model’s calibration was evaluated using the Hosmer-Lemeshow goodness of fit test. Finally, the decision curve analysis (DCA) was performed to reveal the net benefits with each threshold probability.

Records lacking information on age, gender, Scr, blood gas analysis, and surgical records were excluded from all considerations. In this dataset, less than 5% of the covariates contain missing entries. Due to the low proportion of missing data, imputation was deemed unnecessary, the missing values were treated as “missing” in the data analysis to preserve the original state of the data. Statistical analyses were performed using R 4.0.1 (R Foundation for Statistical Computing, Vienna, Austria) and SPSS 28.0 (IBM Corp, Chicago, IL, United States) software. Statistical significance was accepted at the 0.05 level, and all tests were two-tailed.

## 3 Results

### 3.1 Derivation cohort

**Figure 1:**
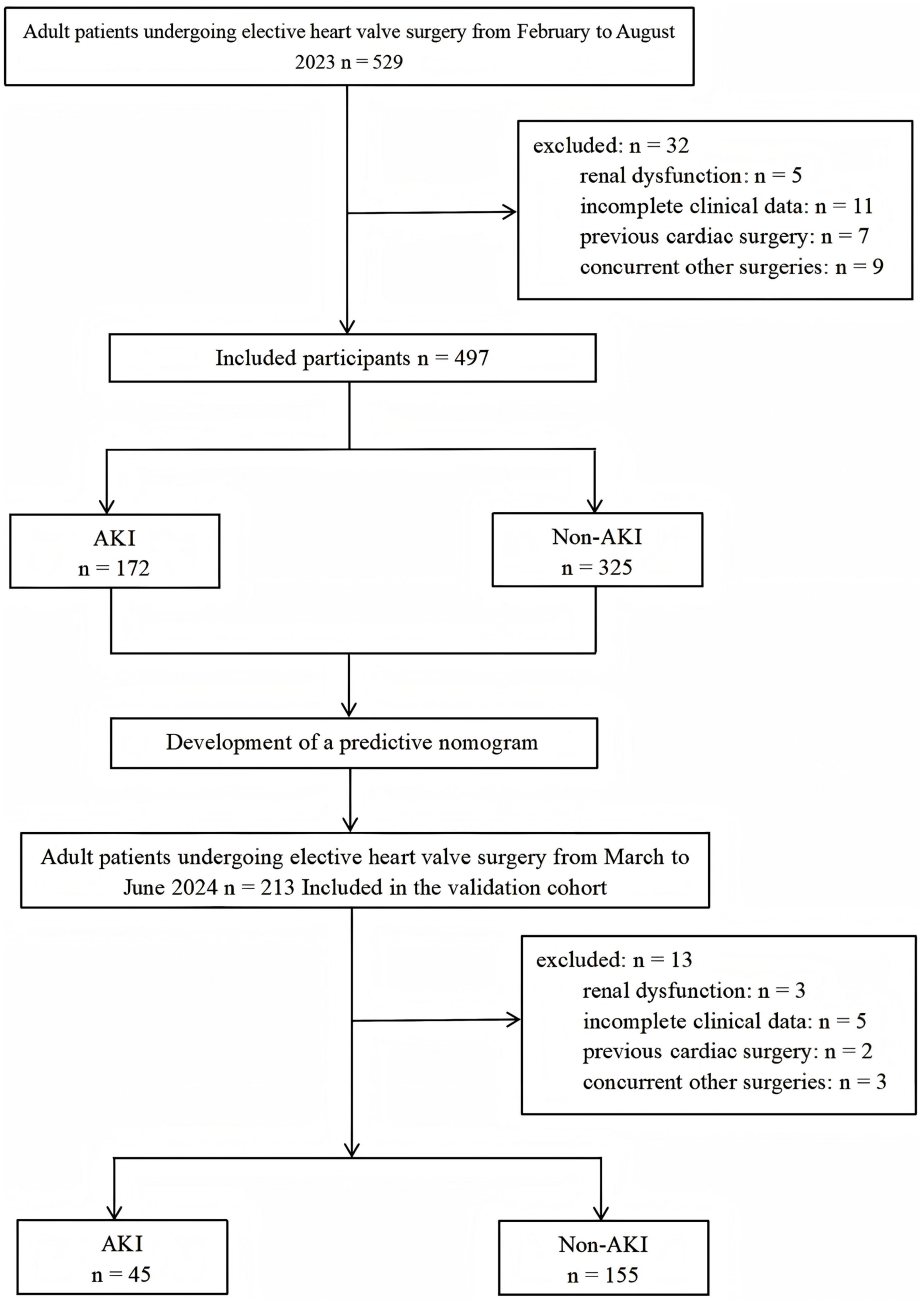
Overall workflow of patient’s enrollment. AKI, acute kidney injury.

Between February and August 2023, total of 529 patients received elective cardiac valve surgery. Among them, 5 patients with preoperative kidney dysfunction, 11 patients with incomplete clinical information, 7 patients with prior cardiac surgery and 9 patients with other concurrent cardiac procedures were eliminated, resulting in an overall exclusion rate of 6.05%. Ultimately, we included only 497 patients in the analysis (Figure 1). By using KDIGO definition of AKI, 172 (34.6%) patients experienced AKI, among which, 132 (76.7%) were stage 1, 21 (12.2%) were stage 2, and 19 (11.0%) were stage 3 AKI. Univariate logistic regression analysis of the derivation cohort is shown in Supplementary Table S1.

The median patient age was 60 years, and there were 268 males (54%) and 229 females (46%). All baseline demographics and concomitant diseases are summarized in Table 1. Relative to non-AKI patients, AKI patients were older [59 (52, 65) vs. 63 (56, 69) years, *p* < 0.001], with higher incidences of hypertension, diabetes and coronary heart disease (CHD) (all *p* < 0.05). Moreover, AKI patients exhibited augmented preoperative Scr, D-Dimer and fibrinogen degradation products (FDP) levels, relative to the non-AKI patients (all *p* < 0.05). In contrast, the hemoglobin (Hb), PLT, Alb and estimated glomerular filtration rate (eGFR) levels were drastically diminished relative to the non-AKI patients (all *p* < 0.05). The proportion of patients using ACEIs/ARBs preoperatively was significantly higher in the AKI group compared to the non-AKI group (*p* = 0.047).

**Table 1:** Preoperative demographic data and clinical characteristics of all patients.

	All patients (*n* = 497)	AKI (*n* = 172)	Non-AKI (*n* = 325)	*p*
Age (years)	60 (53, 66)	63 (56, 69)	59 (52, 65)	<0.001
Age > 65 years, *n* (%)	165 (33.2)	81 (47.1)	84 (25.8)	<0.001
Gender, male, *n* (%)	268 (54.0)	100 (58.1)	168 (51.9)	0.181
BMI (kg/m^2^)	24.5 (22.3, 26.85)	24.65 (22.8, 27.3)	24.5 (22.25, 26.45)	0.31
Hypertension, *n* (%)	107 (21.5)	48 (27.9)	59 (17.9)	0.012
Diabetes, *n* (%)	26 (5.2)	16 (9.3)	10 (3.1)	0.003
Hyperlipidemia, *n* (%)	30 (6.0)	15 (8.7)	15 (4.6)	0.068
CHD, *n* (%)	31 (6.2)	23 (13.4)	8 (2.5)	<0.001
AF, *n* (%)	205 (41.2)	79 (45.9)	126 (38.8)	0.123
LVEF (%)	61 (57, 65)	61 (56, 65)	61 (58, 65)	0.683
Hb (g/L)	13.2 (12.3, 14.2)	13.0 (12.2, 14.1)	13.3 (12.45, 14.3)	0.025
WBC (*10^9^/L)	5.89 (4.98, 6.91)	5.94 (5.03, 7.00)	5.88 (4.96, 6.9)	0.518
PLT (*10^9^/L)	185 (156.5, 218.5)	181 (148, 211.75)	188 (160, 222.5)	0.044
Alb (g/dL)	43.2 (40.8, 45.5)	42.6 (40, 44.78)	43.5 (41.2, 45.9)	0.001
Scr (μmol/L)	76.5 (66.85, 87.75)	80.65 (67.75, 92.58)	75.2 (65.4, 85.65)	0.001
eGFR (mL/min/1.73m^2^)	88.32 (74.92, 97.63)	83.01 (69.95, 93.73)	90.39 (78.46, 98.82)	<0.001
PT (sec)	11.6 (11.1, 12.5)	11.7 (11.1, 13.1)	11.5 (11.1, 12.3)	0.055
APTT (sec)	31.4 (29.7, 33.6)	31.7 (29.7, 34.4)	31.25 (29.7, 33.58)	0.214
D-Dimer (ng/mL)	80.5 (47, 149.25)	109 (60.75, 219.25)	71.5 (42.75, 130.5)	<0.001
FDP (μg/mL)	0.73 (0.5, 1.2)	0.9 (0.5, 1.53)	0.7 (0.4, 1.1)	<0.001
Diuretics, *n* (%)	432 (86.9)	155 (90.1)	277 (85.2)	0.124
Use of ACEIs/ARBs, *n* (%)	66 (13.3)	30 (17.4)	36 (11.1)	0.047
Angiography, *n* (%)	271 (55)	93 (54.7)	178 (55.1)	0.932
MCV (fL)	90.6 (88.1, 93.5)	90.4 (87.63, 93.75)	90.7 (88.2, 93.5)	0.758

BMI, body mass index; CHD, coronary heart disease; AF, atrial fibrillation; LVEF, left ventricular ejection fraction; Hb, hemoglobin; WBC, white blood cells; PLT, platelets; Alb, albumin; Scr, serum creatinine; eGFR, estimated glomerular filtration rate; PT, prothrombin time; APTT, activated partial thromboplastin time; FDP, fibrinogen Degradation Products; ACEIs, angiotensin-converting enzyme inhibitors; ARBs, angiotensin receptor blockers; MCV, mean corpuscular volume.

Overall, 70 (14.1%) patients, had mitral valve (MV) surgery, 96 (19.3%) had aortic valve (AV) surgery, 249 (50.1%) had combined mitral and tricuspid valve (TV) surgery, 21 (4.2%) had combined mitral and aortic valve surgery, and 61 (12.3%) had combined mitral, tricuspid and aortic valve surgery. We observed marked differences in surgical interventions between the two cohorts (*p* = 0.014).

Surgery-related details are listed in Table 2. Relative to non-AKI patient, AKI patients experienced prolonged CPB and ACx durations, along with markedly augmented repeat CPB incidences (all *p* < 0.001). During surgery, the AKI patients had a higher ratio of red blood cells (RBC) and plasma transfusions compared to the non-AKI patients (all *p* < 0.05). The intraoperative nadir HCT levels in patients with AKI were significantly lower than those in non-AKI patients (*p* = 0.026), and the VIS were significantly higher in the AKI group compared to the non-AKI group (*p* = 0.001). The continuous variables (such as intraoperative blood loss and intraoperative urine volume) are converted into dichotomous variables based on their medians. When compared to the non-AKI group, patients in the AKI group exhibited a notably higher proportion of intraoperative blood loss exceeding 700 mL and a markedly lower proportion of intraoperative urine volume exceeding 1,200 mL (all *p* < 0.05).

**Table 2:** Clinical features of surgery.

	All patients (*n* = 497)	AKI (*n* = 172)	Non-AKI (*n* = 325)	*p*
Type of surgery, *n* (%)				0.014
MV	70 (14.1)	19 (11)	51 (15.7)	
AV	96 (19.3)	32 (18.6)	64 (19.7)	
MV + TV	249 (50.1)	81 (47.1)	168 (51.7)	
MV + AV	21 (4.2)	7 (4.1)	14 (4.3)	
MV + TV + AV	61 (12.3)	33 (19.2)	28 (8.6)	
Bleeding volume > 700 mL, *n* (%)	265 (53.3)	105 (61)	160 (49.2)	0.012
Urine volume > 1,200 mL, *n* (%)	233 (47)	66 (38.4)	167 (51.5)	0.005
CPB time (min)	123 (100, 153)	133.5 (106, 166)	118 (97, 143)	<0.001
ACx time (min)	87 (68, 113)	96 (71.25, 128)	83 (67.5, 107.5)	<0.001
Repeat CPB, *n* (%)	19 (3.8)	16 (9.3)	3 (0.9)	<0.001*
RBC transfusion, *n* (%)	203 (40.8)	98 (57)	105 (32.3)	<0.001
Plasma transfusion, *n* (%)	155 (31.2)	76 (44.2)	79 (24.3)	<0.001
Nadir HCT (L/L)	39 (37, 43)	39 (37, 42)	40 (37, 43)	0.026
VIS (score)	5 (5, 7)	6 (5, 8)	5 (5, 7)	0.001
Fluid balance (mL)	230 (−350, 710)	300 (−256, 800)	160 (−400, 633)	0.066

MV, mitral valve; AV, aortic valve; TV, tricuspid valve; RBC, red blood cell; CPB, cardiopulmonary bypass; ACx, aortic cross-clamping; HCT, hematocrit; VIS, vasoactive–inotropic score. *Fisher’s exact probability method.

Postoperative complications and prognostic data are summarized in Table 3. Relative to the non-AKI patients, AKI patients experienced substantially prolonged ICU and hospital stays (all *p* < 0.001). The postoperative kidney replacement therapy, complication, and in-hospital mortality rates were also higher among the AKI versus non-AKI patients, suggesting that AKI patients experience significantly worse outcome.

**Table 3:** Postoperative complications and outcomes.

	All patients (*n* = 497)	AKI (*n* = 172)	Non-AKI (*n* = 325)	*p*
Pulmonary infection, *n* (%)	21 (4.2)	20 (11.6)	1 (0.3)	<0.001*
Hypovolemia, *n* (%)	75 (15.1)	24 (14)	51 (15.7)	0.606
Anemia, *n* (%)	98 (19.7)	46 (26.7)	52 (16.0)	0.004
Hypoalbuminema, *n* (%)	160 (32.2)	61 (35.5)	99 (30.5)	0.256
Thrombopenia, *n* (%)	6 (1.2)	5 (2.9)	1 (0.3)	0.021*
Re-exploration, *n* (%)	13 (2.6)	11 (6.4)	2 (0.6)	<0.001*
In-hospital death, *n* (%)	9 (1.8)	9 (5.2)	0 (0)	<0.001*
KRT, *n* (%)	17 (3.4)	17 (9.9)	0 (0)	<0.001*
Hospital stay, (d)	6 (5, 7)	7 (5, 9)	5 (5, 7)	<0.001
ICU duration, (h)	21 (16, 40)	32.5 (19.13, 68)	20 (15, 25)	<0.001

KRT, kidney replacement therapy; ICU, intensive care unit; *Fisher’s exact probability method.

We further conducted subgroup analyses, stratifying patients according to the severity of AKI based on the KDIGO criteria. The comparative data among the subgroups are presented in Supplementary Table S2. Variables with *p* < 0.05 in the univariate analysis were included in the multivariate analysis, followed by a multivariate ordinal logistic regression analysis. Supplementary Table S3 lists the independent risk factors influencing the severity of AKI after cardiac valve surgery.

### 3.2 Development of a predictive nomogram

**Figure 2:**
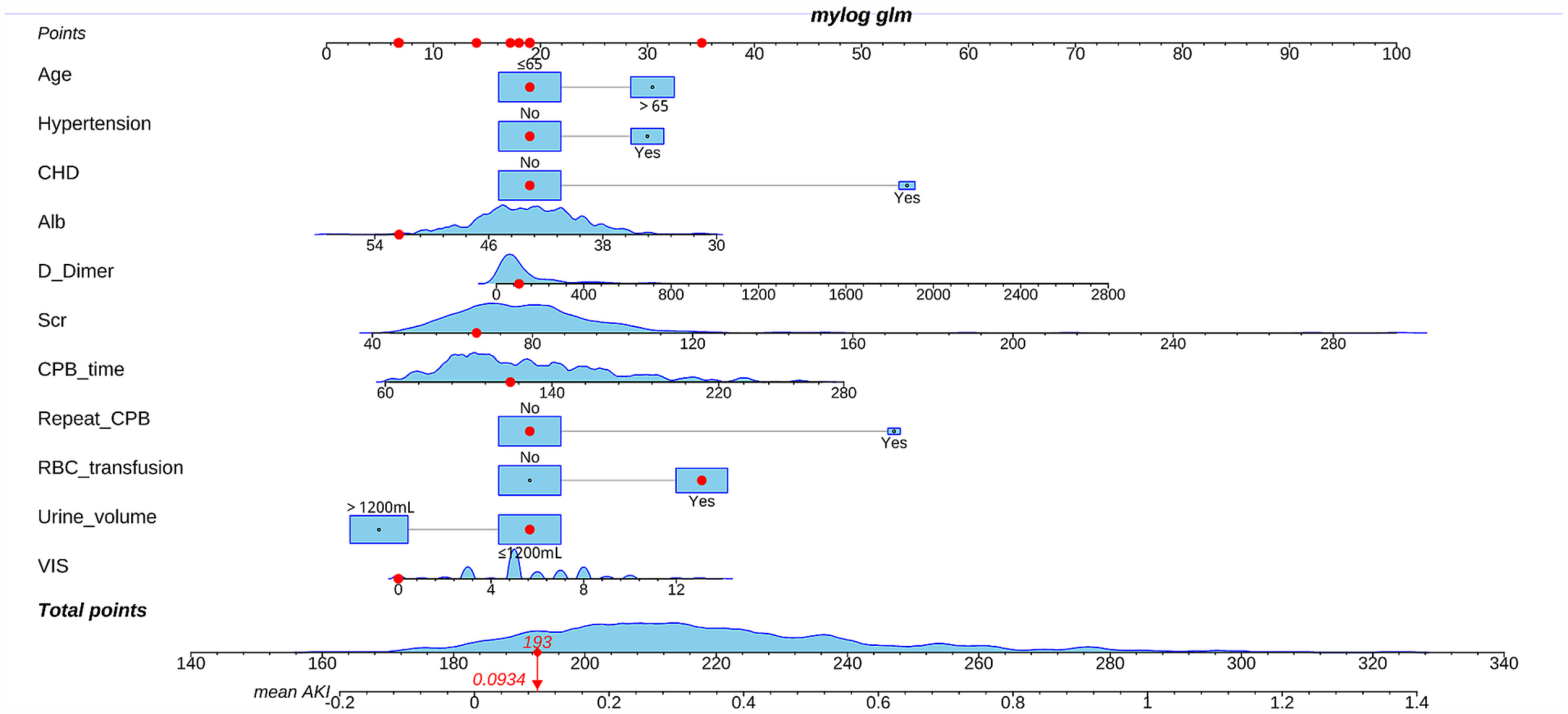
Nomogram of the prediction model predicting the rate acute kidney injury (AKI) following cardiac valve surgery.

Pre- and intraoperative variables that achieved *p* < 0.05 in univariate analysis were entered into multivariate analysis. We adjusted for confounders, such as, gender and BMI. Subsequent binary logistic stepwise regression analysis revealed independent postoperative AKI risk factors to be advanced age (OR: 1.855; 95%CI: 1.154–2.982; *p* = 0.011), preoperative hypertension (OR: 1.91; 95%CI: 1.122–3.252; *p* = 0.017), CHD (OR: 6.773; 95%CI: 2.591–17.702; *p* < 0.001), preoperative Alb (OR: 0.924; 95%CI: 0.866–0.985; *p* = 0.015), D-Dimer (OR: 1.001; 95%CI: 1.000–1.002; *p* = 0.038), Scr (OR: 1.025; 95%CI: 1.010–1.039; *p* = 0.001), CPB duration (OR: 1.011; 95%CI: 1.005–1.018; *p* = 0.001), repeat CPB (OR: 6.195; 95%CI: 1.546–24.829; *p* = 0.010), intraoperative RBC transfusion (OR: 2.560; 95%CI: 1.596–4.107; *p* < 0.001), urine volume > 1,200 mL (OR: 0.406; 95%CI: 0.251–0.656; *p* < 0.001) and VIS (OR: 1.135; 95%CI: 1.032–1.248; *p* = 0.009) (Table 4). All risk factor OR values was >1; whereas, all single protective factor OR value was <1. Using the aforementioned information, we next constructed a high-risk prediction nomogram model (Figure 2). Subsequently, we plan to embed the nomogram algorithm into the electronic health record system to automatically extract information from patient data and generate risk assessment results, thereby applying the predictive model in clinical practice to enhance clinical work efficiency.

**Table 4:** Multivariate conditional logistic stepwise regression analysis for AKI.

	*β*	*W*	OR	95% CI	*p* value
Age > 65 years	0.618	6.501	1.855	1.154–2.982	0.011
Hypertension	0.647	5.676	1.910	1.122–3.252	0.017
CHD	1.913	15.23	6.773	2.591–17.702	<0.001
Alb	−0.079	5.899	0.924	0.866–0.985	0.015
D-Dimer	0.001	4.321	1.001	1.000–1.002	0.038
Scr	0.024	11.648	1.025	1.010–1.039	0.001
CPB time	0.011	11.123	1.011	1.005–1.018	0.001
Repeat CPB	1.824	6.630	6.195	1.546–24.829	0.010
RBC transfusion	0.940	15.20	2.560	1.596–4.107	<0.001
Urine volume > 1,200 mL	−0.902	13.535	0.406	0.251–0.656	<0.001
VIS	0.126	6.771	1.135	1.032–1.248	0.009

OR, odds ratio; CI, confidence interval; CHD, coronary heart disease; Alb, albumin; eGFR, estimated glomerular filtration rate; CPB, cardiopulmonary bypass; VIS, vasoactive–inotropic score.

The employed logic regression equation was as follows: logit *p* = 0.618 × advanced age + 0.647 × Hypertension +1.913 × CHD − 0.079 × Alb +0.001 × D-Dimer +0.024 × Scr + 0.011 × CPB time + 1.824 × repeat CPB + 0.94 × RBC transfusion − 0.902 × urine volume > 1,200 mL + 0.126 × VIS − 2.076.

### 3.3 Validation cohort

From March to June 2024, total of 213 patients were screened according to the inclusion criteria, with 3 patients excluded due to preoperative kidney insufficiency, 5 due to incomplete clinical data, 2 patients with prior cardiac surgery and 3 patients with other concurrent cardiac procedures were eliminated, resulting in an overall exclusion rate of 6.10%. Ultimately, 200 patients were included in the validation cohort for analysis. By using KDIGO definition of AKI, 45 (22.5%) patients developed AKI and 155 (77.5%) patients did not. The median age was 58 years, and 119 patients (59.5%) were male. Similar to the derivation cohort, Compared with patients in the non-AKI group, patients in the AKI group had a higher incidence of preoperative CHD and atrial fibrillation (AF). Preoperative D-Dimer levels in AKI patients were significantly higher than those in non-AKI patients, whereas preoperative PLT and eGFR levels were significantly lower than those in non-AKI patients. The probability of RBC and plasma transfusion, intraoperative blood loss, CPB time and VIS in AKI group were significantly higher than those in non-AKI group (Supplementary Table S4). Comparisons between the derivation and validation cohorts can be found in Supplementary Table S5.

### 3.4 Validation of the nomogram

**Figure 3:**
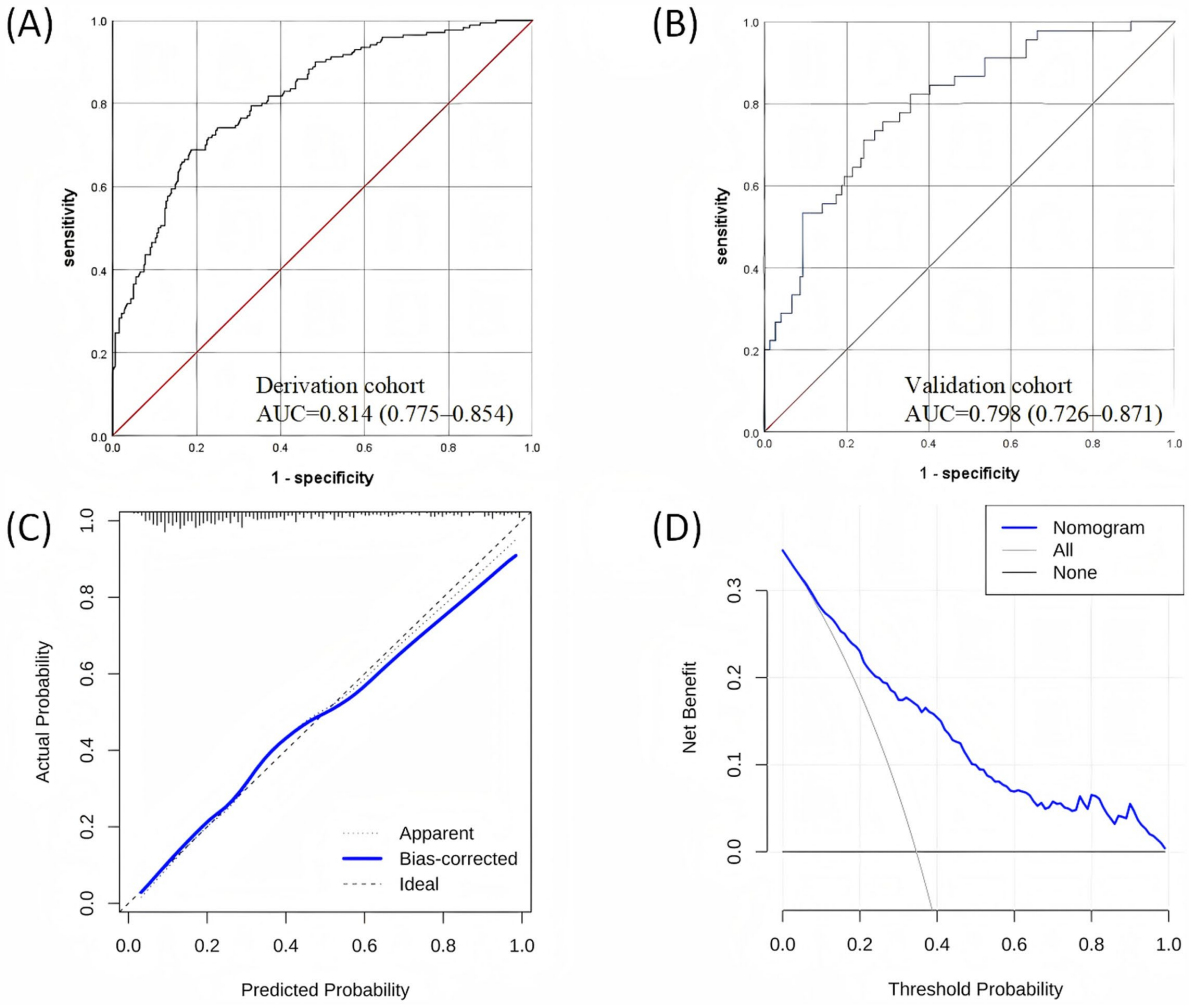
Validation of the nomogram: **(A)** ROC curve in the derivation dataset; **(B)** ROC curve in the validation dataset; **(C)** Calibration curve for the derivation dataset; **(D)** Decision curve analysis for the derivation dataset. ROC, receiver operating characteristic.

The nomogram had a satisfactory capacity with the areas under ROC curve (AUC) of 0.814 (95%CI: 0.775–0.854) and 0.798 (95%CI: 0.726–0.871) in the derivation and validation cohorts, respectively (Figures 3A,B). Besides, the calibration plots represented an excellent agreement between actual observations and model prediction (Figure 3C). The DCA curve indicated that using the new model for risk assessment of postoperative AKI generated a net benefit within most of the range of prediction thresholds (Figure 3D).

## 4 Discussion

Herein, we demonstrated that the post-cardiac valve surgery AKI incidence was 34.6%, which closely similar to earlier reports (35). We further revealed that AKI patients experienced prolonged ICU and hospital stays, with augmented postoperative complications and in-hospital mortality rates, which severely impacted patient outcome. AKI treatment strategies are generally limited, and rely primarily on symptom management, before addressing underlying causes (36). Hence, early identification and prevention is critical for decreasing AKI risk and enhancing patient outcome.

Currently, risk factors associated with the development of CSA-AKI have been well-studied, and related predictive models have been established. These models are instrumental in identifying patients at high risk for preoperative CSA-AKI. In 2007, Wijeysundera et al. (17) developed the SRI model (SRI score, Toronto). The retrospective cohort study derived and validated a simple and accurate predictor for RRT following cardiac surgery. The AUC for the derivation, Toronto validation, and Ottawa validation cohorts were 0.81, 0.78, and 0.78, respectively. The MCSPI research team has developed a risk score based on a study involving 4,801 patients who underwent CABG with CPB at 70 centers across 16 countries (18). The study identified the correlation between predictor variables and kidney composite events (kidney insufficiency and/or kidney failure) following cardiac surgery. The risk index was then evaluated in a validation cohort of 2,420 patients. The model demonstrated an AUC of 0.84 in the derivation set and an AUC of 0.8 in the validation cohort. The AKICS score is derived from a prospective study that included 603 patients undergoing elective CABG and/or valve replacement surgery (19). The study developed a predictive score for AKI following cardiac surgery. The AKICS score demonstrated a discriminatory AUC of 0.843 in the derivation cohort. When applied to an independent, prospectively followed validation cohort of 215 patients undergoing cardiac surgery at the same institution, the AKICS score showed good discriminatory performance with an AUC of 0.847. However, existing risk prediction tools have certain limitations. They have demonstrated good discrimination in foreign populations (37) but only average performance in Chinese populations (38). A retrospective study involving Chinese patients revealed low discriminative ability of AKICS in predicting CSA-AKI (AUC = 0.610). Similarly, for predicting RRT-AKI, the discrimination of Cleveland score (AUC = 0.684), Mehta score (AUC = 0.708), and SRI score (AUC = 0.622) were not good (39). Therefore, there is a need to develop a prediction model for AKI after heart valve surgery that is more suitable for the Chinese population (40).

Our study exclusively enrolled adult patients undergoing cardiac valve surgery, with the primary outcome being the occurrence of postoperative AKI. We focused primarily on preoperative and intraoperative indicators to identify risk factors for AKI, such as advanced age, comorbid cardiovascular diseases, CPB duration and blood transfusion (41). In addition, we revealed independent AKI risk factors post-cardiac valve surgery, such as preoperative albumin level, intraoperative urine output, VIS and repeat CPB development. Our study established and externally validated a predictive model for AKI after cardiac valve surgery, with AUCs of 0.814 and 0.798 in the derivation and validation groups, respectively. The predictive model demonstrated good discrimination and calibration in both the derivation and validation cohorts, providing a theoretical basis for early prevention and perioperative management of AKI.

There is still controversy over the potential link between preoperative CHD and postoperative AKI risk. Several studies reported a marked link between moderate to severe CHD combined with valve surgery and late postoperative AKI. Furthermore, severe CHD is associated with longer ICU stays and higher in-hospital mortality rates (42). In contrast, other studies revealed CHD as a protective factor for AKI occurrence likely due to the fact that CHD patients receive extra care prior to surgery (43). In this report, we demonstrated that the postoperative AKI risk rose by 7-fold among patients with preoperative combined coronary artery atherosclerotic heart disease. Among coronary artery disease patients, preoperative coronary angiography is essential for severity assessment. Unfortunately, contrast agents used in such procedures may cause kidney injury due to direct toxicity, kidney medullary hypoxia, oxidative stress, apoptosis and inflammation (44, 45). Moreover, aberrations in lipid metabolism not only influence coronary arteries but also impact systemic vessels, such as, kidney arteries. One study revealed that coronary artery disease patients are more susceptible to kidney artery stenosis. Moreover, the CHD severity is independently linked to kidney artery stenosis (46). Kidney artery stenosis that exceeds 50–60% can severely impact kidney blood flow, thereby causing significant dysfunction in kidney perfusion (47).

We also found that the preoperative kidney and coagulation activity were intricately linked to postoperative AKI. Moreover, the preoperative albumin level served as a protective factor against postoperative AKI development. For each 1 g/dL rise in albumin content, the AKI incidence reduced to 0.92 folds. Earlier reports suggested that the preoperative albumin concentration may be associated with poor nutritional status and chronic inflammation among AKI patients (48). Albumin is a major plasma protein that contributes to 70–80% of the plasma oncotic pressure. It increases the effective circulating volume by accelerating the effective reabsorption of fluid accumulation from the interstitial space, which augments kidney flow and urine output (49). Apart from the albumin impact on intravascular volume, it also has antioxidant, anticoagulant, anti-inflammatory, and antiapoptotic effects (50). Albumin possesses remarkable beneficial effect on kidney function that is mediated using specific networks, namely, S-nitrosoalbumin formation via reaction with nitrogen oxides, alongside kidney perfusion and glomerular filtration maintenance via kidney vessel vasodilation (51). It also protects kidney tubules by expelling reactive oxygen species, thereby eliminating oxidative damage, and it binds and delivers protective phospholipids (52). The albumin-mediated anticoagulant and antithrombotic properties utilize albumin interaction with nitric oxide free radicals, for example, S-nitrosothiols to suppress their rapid inactivation and facilitate a prolonged antiplatelet aggregation effect (53). Prior studies revealed that a reduced preoperative circulating albumin concentration can enhance postoperative AKI risk, kidney replacement therapy rate (54), and mortality (55) among patients undergoing cardiac surgery. Furthermore, prolonged ICU and hospital stays are often observed (56), leading to poor prognosis for these patients (57). Till date, there is some controversy whether perioperative exogenous albumin supplementation improves postoperative kidney activity (58). However, studies have shown that the use of human serum albumin as a priming solution in the CPB circuit has beneficial effects on kidney blood flow and microcirculation (59). A retrospective study has shown that albumin as an alternative in cardiopulmonary bypass surgery is safe and effective for patients with increased bleeding or kidney failure (60). Hosseinzadeh Maliki et al. found that patients who received 25 grams of albumin (500 mL of 5% albumin) in the bypass circuit priming solution experienced a lower increase in postoperative Scr and a smaller decrease in eGFR compared to patients who received Hydroxyl Ethyl Starch (HES) (61).

AKI pathogenesis and related signaling networks are relatively complicated and diverse. In addition, AKI can develop at any point during the perioperative period. Herein, we revealed that the intraoperative RBC transfusion augments postoperative AKI incidence by 2.56 fold, strongly corroborating earlier data (62). This effect may be the result of loss of red blood cell deformability during storage, ATP and 2,3-diphosphoglycerate depletion, enhanced adhesion to vascular endothelium (63), proinflammatory molecule aggregation and procoagulant lipid release (64). Inflammatory activation via allogeneic blood products, exacerbates tissue oxidative stress, produces hypoxia, and accelerates organ dysfunction or failure (65). Moreover, red blood cells undergo irreversible morphological and biochemical alterations during storage, which directly damage kidney tubules and impact glomerular filtration function (66). Therefore, demonstrated that prolonged CPB duration was a independent risk factor for postoperative AKI. Moreover, the postoperative AKI incidences rose 6-folds among patients who repeated CPB during surgery. CPB is associated with marked inflammation and hemodilution, which can result in insufficient kidney perfusion (67), particularly, within the highly oxygen-dependent medullary region (68), which is critical for AKI pathogenesis. In addition, ischemia–reperfusion injury and reduced cardiac output induce tissue hypoxia and endothelial damage, which exacerbate the present condition (69).

Multiple studies have identified critical perioperative independent risk factors for AKI development. Modifiable risk factors require active management, including the proactive underlying disease intervention, along with improved intraoperative management. In case of high-risk patients, postoperative kidney function must be closely monitored. Early prevention and timely diagnosis are essential in reducing AKI incidence and enhancing patient clinical outcomes.

Our study has several limitations. Firstly, this study is a single-center, retrospective study, and therefore, the risk of bias cannot be overlooked when interpreting the results. External validation of the model at other centers is necessary for its widespread application in the future. Secondly, the sample size in this study is limited, which constrains the statistical power for subgroup analyses. Future validation of the current study’s findings in large-scale population studies is required. Thirdly, due to the lack of data regarding patients’ preoperative hydration status and the type of anesthetic agents used in the electronic medical records, we were unable to include them in the analysis. Lastly, this report lacked data on long-term patient outcomes (e.g., 1-year mortality) and survival analysis. Given these limitations, we recommend a long-term follow-up study to examine the influence of postoperative AKI on long-term patient survival following cardiac valve surgery.

## 5 Conclusion

Our data confirmed the substantial AKI risk following cardiac valve surgery, and its negative impact on patient outcome. Using logistic regression analysis, we also identified several independent pre- and intraoperative risk factors, which were later used to generate a predictive nomogram model with enhanced accuracy and sensitivity. Together, these findings provide a strong theoretical foundation of AKI prediction following cardiac valve surgery and for intraoperative management.

## Data Availability

The raw data supporting the conclusions of this article will be made available by the authors, without undue reservation.
