# Supplementary material for: Development and validation of a prediction model for acute kidney injury following cardiac valve surgery
Source: Front Med (Lausanne). 2025 Jan 31;12:1528147. doi: 10.3389/fmed.2025.1528147 (PMC11825392; doi:10.3389/fmed.2025.1528147)
Supplement: Supplementary file 1 [file Table_1.DOCX]

**Supplementary Table 1.** Univariate logistic regression analysis of AKI in derivation cohort

|  | ***β*** | ***W*** | **OR** | **95% CI** | ***P* value** |
| --- | --- | --- | --- | --- | --- |
| Age (years) | 0.046 | 20.663 | 1.047 | 1.026-1.068 | <0.001 |
| Age>65years | 0.938 | 22.317 | 2.554 | 1.731-3.768 | <0.001 |
| Gender, male | 0.254 | 1.785 | 1.290 | 0.888-1.873 | 0.182 |
| BMI (kg/m^2^) | 0.020 | 0.653 | 1.021 | 0.971-1.073 | 0.419 |
| Hypertension | 0.557 | 6.251 | 1.745 | 1.128-2.700 | 0.012 |
| Diabetes | 1.173 | 7.992 | 3.231 | 1.433-7.285 | 0.005 |
| Hyperlipidemia | 0.680 | 3.238 | 1.975 | 0.941-4.143 | 0.072 |
| CHD | 1.811 | 18.390 | 6.117 | 2.673-13.995 | <0.001 |
| AF | 0.294 | 2.374 | 1.342 | 0.923-1.950 | 0.123 |
| LVEF (%) | -0.018 | 1.468 | 0.982 | 0.954-1.011 | 0.226 |
| Hb (g/L) | -0.130 | 3.778 | 0.878 | 0.770-1.001 | 0.052 |
| WBC (*10^9^/L) | 0.053 | 0.847 | 1.055 | 0.941-1.182 | 0.357 |
| PLT (*10^9^/L) | -0.003 | 2.454 | 0.997 | 0.993-1.001 | 0.117 |
| Alb (g/dL) | -0.108 | 16.124 | 0.897 | 0.851-0.946 | <0.001 |
| Scr (μmol/L) | 0.022 | 15.506 | 1.022 | 1.011-1.033 | <0.001 |
| eGFR (mL/min/1.73m^2^) | -0.025 | 20.864 | 0.975 | 0.964-0.986 | <0.001 |
| PT (sec) | 0.025 | 1.627 | 1.025 | 0.987-1.065 | 0.202 |
| APTT (sec) | 0.030 | 2.358 | 1.031 | 0.992-1.072 | 0.125 |
| D-Dimer (ng/mL) | 0.002 | 12.105 | 1.002 | 1.001-1.003 | 0.001 |
| FDP (μg/mL) | 0.298 | 13.651 | 1.347 | 1.150-1.578 | <0.001 |
| diuretics | 0.457 | 2.332 | 1.580 | 0.878-2.842 | 0.127 |
| Use of ACEIs/ARBs | 0.528 | 3.897 | 1.696 | 1.004-2.866 | 0.048 |
| angiography | -0.028 | 0.022 | 0.972 | 0.671-1.409 | 0.882 |
| MCV (fL) | 0.000 | 0.000 | 1.000 | 0.961-1.041 | 0.999 |
| Type of surgery |  |  |  |  |  |
| MV |  | 11.970 |  |  | 0.018 |
| AV | 0.294 | 0.727 | 1.342 | 0.682-2.64 | 0.934 |
| MV+TV | 0.258 | 0.734 | 1.294 | 0.718-2.334 | 0.391 |
| MV+AV | 0.294 | 0.302 | 1.342 | 0.470-3.832 | 0.583 |
| MV+TV+AV | 1.152 | 9.594 | 3.164 | 1.526-6.557 | 0.002 |
| bleeding volum>700mL | 0.480 | 6.269 | 1.616 | 1.110-2.353 | 0.012 |
| urine volume>1200mL | -0.536 | 7.763 | 0.585 | 0.402-0.853 | 0.005 |
| CPB time (min) | 0.014 | 28.348 | 1.014 | 1.009-1.019 | <0.001 |
| ACx time (min) | 0.012 | 17.870 | 1.012 | 1.007-1.018 | <0.001 |
| repeat CPB | 0.399 | 14.194 | 11.009 | 3.161-38.341 | <0.001 |
| RBC transfusion | 1.021 | 27.564 | 2.775 | 1.896-4.062 | <0.001 |
| plasma transfusion | 0.902 | 20.202 | 2.465 | 1.663-3.654 | <0.001 |
| nadir HCT (L/L) | -0.042 | 3.636 | 0.958 | 0.917-1.001 | 0.057 |
| VIS (score) | 0.131 | 11.071 | 1.140 | 1.055-1.232 | 0.001 |
| fluid balance (mL) | 0.000 | 0.000 | 1.000 | 1.000-1.000 | 0.074 |

**Abbreviations:** OR: odds ratio; CI: confidence interval; CHD: coronary heart disease; Alb: albumin; eGFR: estimated glomerular filtration rate; CPB: cardiopulmonary bypas; VIS: vasoactive–inotropic score. BMI: body mass index; CHD: coronary heart disease; AF: atrial fibrillation; LVEF: left ventricular ejection fraction; Hb: hemoglobin; WBC: white blood cells; PLT: platelets; Alb: albumin; Scr: serum creatinine; eGFR: estimated glomerular filtration rate; PT: prothrombin time; APTT: activated partial thromboplastin time; FDP: fibrinogen Degradation Products; ACEIs: angiotensin-converting enzyme inhibitors; ARBs: angiotensin receptor blockers; MCV: mean corpuscular volume; MV: mitral valve; AV: aortic valve; TV: tricuspid valve; RBC: red blood cell; CPB: cardiopulmonary bypass; ACx: aortic cross-clamping; HCT: hematocrit; VIS: vasoactive–inotropic score.

**Supplementary Table 2.** Subgroup analysis of AKI severity

|  | **Non-AKI (n=325)** | **AKI-1 (n=132)** | **AKI-2 (n=21)** | **AKI-3 (n=19)** | ***P* value** |
| --- | --- | --- | --- | --- | --- |
| Age (years) | 59 (52.5,65) | 63 (55,68) | 61 (58,67.5) | 69 (60,73) | <0.001 |
| Age>65years, n (%) | 84 (25.8) | 58 (43.9) | 10 (47.6) | 13 (47.1) | <0.001 |
| Gender, male, n (%) | 167 (51.5) | 83 (62.9) | 10 (47.6) | 8 (42.1) | 0.094 |
| BMI (kg/m^2^) | 24.5 (22.3,26.45) | 24.8 (23.125,27) | 26.6 (21.9,28.7) | 23.2 (20.5,26) | 0.261 |
| Hypertension, n (%) | 59 (18.2) | 38 (28.8) | 5 (23.8) | 5 (26.3) | 0.085 |
| Diabetes, n (%) | 10 (3.1) | 12 (9.1) | 1 (4.8) | 3 (15.8) | 0.009* |
| Hyperlipidemia, n (%) | 15 (4.6) | 10 (7.6) | 3 (14.3) | 2 (10.5) | 0.107* |
| CHD, n (%) | 8 (2.5) | 16 (12.1) | 4 (19) | 3 (15.8) | <0.001 |
| AF, n (%) | 126 (38.8) | 57 (43.2) | 13 (61.9) | 9 (47.4) | 0.17 |
| LVEF (%) | 61 (58,65) | 61 (56,65) | 61 (59,64.5) | 65 (55,68) | 0.597 |
| Hb (g/L) | 13.3 (12.5,14.3) | 13 (12.2,14.1) | 13.1 (12.25,14.2) | 12.9 (12.1,14.1) | 0.194 |
| WBC (*10^9^/L) | 5.85 (4.94,6.89) | 5.905 (5.02,6.85) | 6.33 (5.24,7.08) | 6.36 (4.79,9.33) | 0.738 |
| PLT (*10^9^/L) | 187 (160,222) | 183 (148,211) | 172 (152.5,217.5) | 171 (143,208) | 0.32 |
| Alb (g/dL) | 43.5 (41.2,45.9) | 42.75 (40,45) | 42.2 (38,44.35) | 43.3 (40.9,44.5) | 0.005 |
| Scr (μmol/L) | 75.2 (65.4,85.65) | 79.9 (67.45,90.2) | 84.4 (67.25,96.8) | 83.7 (73.1,105.2) | 0.007 |
| eGFR (mL/min/1.73m^2^) | 90.16 (78.12,98.69) | 88.08 (73.18,96.35) | 80.47 (64.96,90.10) | 71.08 (55.35,89.51) | <0.001 |
| PT (sec) | 11.6 (11.1,12.3) | 11.65 (11.2,13.23) | 11.3 (10.95,12) | 11.9 (11.2,13.3) | 0.105 |
| APTT (sec) | 31.4 (29.7,33.6) | 31.7 (29.48,34.73) | 31 (30.4,32.65) | 31.5 (28.8,38.3) | 0.556 |
| D-Dimer (ng/mL) | 71.5 (42.75,130.75) | 108 (56,216) | 115 (65,232.5) | 137 (64,619) | <0.001 |
| FDP (μg/mL) | 0.7 (0.4,1.1) | 0.9 (0.5,1.5) | 0.8 (0.55,1.4) | 1.0 (0.5,4.2) | 0.001 |
| diuretics, n (%) | 277 (85.2) | 117 (88.6) | 20 (95.2) | 18 (94.7) | 0.326 |
| Use of ACEIs/ARBs, n (%) | 36 (11.1) | 21 (15.9) | 5 (23.8) | 4 (21.1) | 0.159 |
| angiography, n (%) | 177 (54.5) | 71 (53.8) | 11 (52.4) | 12 (63.2) | 0.887 |
| MCV (fL) | 90.7 (88.15,93.5) | 90.4 (87.88,93.65) | 89.9 (86.4,93) | 92.6 (89.5,94.8) | 0.388 |
| Type of surgery, n (%) |  |  |  |  | 0.03* |
| MV | 52 (16) | 12 (9.1) | 3 (14.3) | 3 (15.8) |  |
| AV | 64 (19.7) | 28 (21.2) | 2 (9.5) | 2 (10.5) |  |
| MV+TV | 167 (51.4) | 62 (47) | 8 (38.1) | 12 (63.2) |  |
| MV+AV | 14 (4.3) | 6 (4.5) | 1 (4.8) | 0 (0) |  |
| MV+TV+AV | 28 (8.6) | 24 (18.2) | 7 (33.3) | 2 (10.5) |  |
| bleeding volum>700mL, n (%) | 160 (49.2) | 82 (62.1) | 12 (57.1) | 11 (57.9) | 0.087 |
| urine volume>1200mL, n (%) | 167 (51.5) | 54 (40.9) | 8 (38.1) | 4 (21.1) | 0.015 |
| CPB time (min) | 117 (96.5,143) | 136.5 (106,170.75) | 144 (115.5,186.5) | 130 (100,153) | <0.001 |
| ACx time (min) | 83 (67,106.5) | 99.5 (71.5,129) | 110 (82.5,134) | 86 (66,106) | <0.001 |
| repeat CPB, n (%) | 2 (0.6) | 12 (9.1) | 3 (14.3) | 2 (10.5) | <0.001 |
| RBC transfusion, n (%) | 106 (32.6) | 69 (52.3) | 13 (61.9) | 15 (78.9) | <0.001 |
| plasma transfusion, n (%) | 80 (24.6) | 55 (41.7) | 9 (42.9) | 11 (57.9) | <0.001 |
| nadir hematocrit (L/L) | 40 (37,43) | 39 (37,42) | 39 (36.5,43) | 39 (36,42) | 0.207 |
| VIS (score) | 5 (5,7) | 6 (5,8) | 5 (5,7.5) | 5 (5,7) | 0.009 |
| fluid balance (mL) | 200 (-400,647.5) | 314 (-263.75,800) | 126 (-600,980) | 263 (-113,900) | 0.339 |

**Abbreviations:** BMI: body mass index; CHD: coronary heart disease; AF: atrial fibrillation; LVEF: left ventricular ejection fraction; Hb: hemoglobin; WBC: white blood cells; PLT: platelets; Alb: albumin; Scr: serum creatinine; eGFR: estimated glomerular filtration rate; PT: prothrombin time; APTT: activated partial thromboplastin time; FDP: fibrinogen Degradation Products; ACEIs: angiotensin-converting enzyme inhibitors; ARBs: angiotensin receptor blockers; MCV: mean corpuscular volume; MV: mitral valve; AV: aortic valve; TV: tricuspid valve; RBC: red blood cell; CPB: cardiopulmonary bypass; ACx: aortic cross-clamping; HCT: hematocrit; VIS: vasoactive–inotropic score. *Fisher’s exact probability method.

**Supplementary Table 3.** Multiple ordered logistic regression analysis of AKI severity

|  | ***β*** | ***W*** | ***P* value** | **95% CI** |
| --- | --- | --- | --- | --- |
| Age>65years | 0.674 | 93.99 | 0.002 | 0.243-1.105 |
| CHD | 1.605 | 18.195 | <0.001 | 0.868-2.342 |
| Alb | -0.061 | 4.321 | 0.038 | -0.119- -0.003 |
| Scr | 0.018 | 17.222 | <0.001 | 0.009-0.026 |
| D-Dimer | 0.001 | 8.125 | 0.004 | 0.000-0.002 |
| Urine volume>1200mL | -0.886 | 15.722 | <0.001 | -1.324- -0.448 |
| Repeat CPB | 1.821 | 12.085 | 0.001 | 0.794-2.848 |
| RBC transfusion | 0.824 | 11.967 | 0.001 | 0.357-1.291 |

**Abbreviations:** OR: odds ratio; CI: confidence interval; CHD: coronary heart disease; Alb: albumin; Scr: serum creatinine; RBC: red blood cell; CPB: cardiopulmonary bypass.

**Supplementary Table 4.** Baseline characteristics of AKI group and non-AKI group in derivation cohort

|  | **All patients (n=200)** | **AKI (n=45)** | **Non-AKI (n=155)** | ***P* value** |
| --- | --- | --- | --- | --- |
| Age (years) | 58 (50.25,64.75) | 59 (54,65) | 57 (49.75,57) | 0.133 |
| Age>65years, n (%) | 50 (25) | 12 (26.7) | 38 (24.5) | 0.769 |
| Gender, male, n (%) | 119 (59..5) | 22 (48.9) | 97 (62.6) | 0.1 |
| BMI (kg/m^2^) | 24.82±3.50 | 24.74±3.74 | 24.81±3.45 | 0.975 |
| Hypertension, n (%) | 51 (25.5) | 14 (31.1) | 37 (23.9) | 0.327 |
| Diabetes, n (%) | 14 (7) | 5 (11.1) | 9 (5.8) | 0.22 |
| Hyperlipidemia, n (%) | 7 (3.5) | 4 (8.9) | 3 (1.9) | 0.025 |
| CHD, n (%) | 14 (7) | 7 (15.6 ) | 7 (4.5) | 0.011 |
| AF, n (%) | 64 (32) | 21 (46.7) | 43 (27.7) | 0.017 |
| LVEF (%) | 62 (58,65) | 60.5 (56,64) | 62 (58,66) | 0.138 |
| Hb (g/L) | 13.37±1.43 | 13.27±1.78 | 13.39±1.32 | 0.60 |
| WBC (*10^9^/L) | 6.01±1.49 | 5.98±1.62 | 6.01±1.45 | 0.897 |
| PLT (*10^9^/L) | 190.68±55.31 | 171.44±52.76 | 196.26±54.93 | 0.008 |
| Alb (g/dL) | 42.78±3.58 | 41.99±4.38 | 43.01±3.29 | 0.093 |
| Scr (μmol/L) | 76.85 (66.68,88.53) | 77.6 (70.75,94.93) | 76.95 (65.95,88.08) | 0.146 |
| eGFR (mL/min/1.73m^2^) | 89.465 (74.44,100.38) | 76.355 (64.9,96.79) | 91.98 (78.71,101.67) | 0.001 |
| PT (sec) | 11.4 (11.0,12.1) | 11.5 (11.1,12.43) | 11.35 (11.0,12.03) | 0.134 |
| APTT (sec) | 30.9 (29.3,33.1) | 30.8 (28.88,33.08) | 31.2 (29.5,33.2) | 0.805 |
| D-Dimer (ng/mL) | 76 (41,129.25) | 91.5 (49.75,188.75) | 71 (37.75,125.25) | 0.036 |
| FDP (μg/mL) | 0.7 (0.4,1.0) | 0.7 (0.425,1.2) | 0.7 (0.4,1.0) | 0.488 |
| diuretics, n (%) | 184 (92) | 44 (97.8) | 140 (90.3) | 0.105 |
| Use of ACEIs/ARBs, n (%) | 28 (14) | 4 (8.9) | 24 (15.5) | 0.262 |
| angiography, n (%) | 107 (53.5) | 26 (57.8) | 81 (52.3) | 0.513 |
| MCV (fL) | 90.5 (88.13,93.2) | 91.1 (88.7 93.45) | 89.7 (87.78,93.2) | 0.16 |
| Type of surgery, n (%) |  |  |  | 0.261 |
| MV | 24 (12) | 4 (8.9) | 20 (12.9) |  |
| AV | 45 (22.5) | 6 (13.3) | 39 (25.2) |  |
| MV+TV | 102 (51) | 25 (55.6) | 77 (49.7) |  |
| MV+AV | 9 (4.5) | 3 (6.7) | 6 (3.9) |  |
| MV+TV+AV | 20 (10) | 7 (15.6) | 13 (8.4) |  |
| bleeding volum>700mL, n (%) | 103 (51.5) | 29 (64.4) | 74 (47.7) | 0.048 |
| urine volume>1200mL, n (%) | 109 (54.5) | 27 (60) | 82 (52.9) | 0.4 |
| CPB time (min) | 119 (98.75,145.25) | 145 (120.75,176.75) | 112.5 (94,137) | <0.001 |
| ACx time (min) | 86 (70,113) | 106 (91.25,129) | 82 (68,105.25) | <0.001 |
| repeat CPB, n (%) | 6 (3) | 6 (13.3) | 0 (0) | <0.001 |
| RBC transfusion, n (%) | 57 (28.5) | 24 (53.3) | 33 (21.3) | <0.001 |
| plasma transfusion, n (%) | 38 (19) | 18 (40) | 20 (12.9) | <0.001 |
| nadir hematocrit (L/L) | 40 (37,43) | 40 (37,43) | 40 (38,43) | 0.761 |
| VIS (score) | 5 (5,7) | 6 (5,8) | 5 (5,6) | 0.001 |
| fluid balance (mL) | 42.5 (-367.75,588) | -177 (-547.75,450) | 134 (-350,630.5) | 0.048 |

**Abbreviations:** BMI: body mass index; CHD: coronary heart disease; AF: atrial fibrillation; LVEF: left ventricular ejection fraction; Hb: hemoglobin; WBC: white blood cells; PLT: platelets; Alb: albumin; Scr: serum creatinine; eGFR: estimated glomerular filtration rate; PT: prothrombin time; APTT: activated partial thromboplastin time; FDP: fibrinogen Degradation Products; ACEIs: angiotensin-converting enzyme inhibitors; ARBs: angiotensin receptor blockers; MCV: mean corpuscular volume; MV: mitral valve; AV: aortic valve; TV: tricuspid valve; RBC: red blood cell; CPB: cardiopulmonary bypass; ACx: aortic cross-clamping; HCT: hematocrit; VIS: vasoactive–inotropic score.

**Supplementary Table 5.** Baseline characteristics of the populations studied in two groups, the derivation cohort and the validation cohort

|  | **Derivation cohort (n=497)** | **Validation cohort (n=200)** | **P value** |
| --- | --- | --- | --- |
| AKI, n (%) | 172 (34.6) | 45 (22.5) | 0002 |
| Age (years) | 60 (53,66) | 58 (50.25,64.75) | 0.008 |
| Age>65years, n (%) | 165 (33.2) | 50 (25) | 0.034 |
| Gender, male, n (%) | 268 (54) | 119 (59.5) | 0.189 |
| BMI (kg/m^2^) | 24.5 (22.3,26.85) | 24.90 (22.31,27.29) | 0.653 |
| Hypertension, n (%) | 107 (21.5) | 51 (25.5) | 0.257 |
| Diabetes, n (%) | 26 (5.2) | 14 (7) | 0.364 |
| Hyperlipidemia, n (%) | 30 (6) | 7 (3.5) | 0.177 |
| CHD, n (%) | 31 (6.2) | 14 (7) | 0.711 |
| AF, n (%) | 205 (41.2) | 64 (32) | 0.023 |
| LVEF (%) | 61 (57,65) | 62 (58,65) | 0.129 |
| Hb (g/L) | 13.2 (12.35,14.2) | 13.4 (12.5,14.4) | 0.181 |
| WBC (*10^9^/L) | 5.89 (4.98,6.91) | 6.01 (4.94,6.94) | 0.684 |
| PLT (*10^9^/L) | 185 (156.5,218.5) | 185 (153,221.25) | 0.896 |
| Alb (g/dL) | 43.15±3.71 | 42.78±3.58 | 0.226 |
| Scr (μmol/L) | 76.5 (66.85,87.75) | 76.85 (66.675,88.525) | 0.785 |
| eGFR (mL/min/1.73m^2^) | 88.32(74.92,97.63) | 89.465 (74.442,100.38) | 0.161 |
| PT (sec) | 11.6 (11.1,12.5) | 11.4 (11.0,12.1) | 0.008 |
| APTT (sec) | 31.4 (29.7,33.6) | 30.9 (29.3,33.1) | 0.125 |
| D-Dimer (ng/mL) | 80.5 (47,149.25) | 76 (41,129.25) | 0.135 |
| FDP (μg/mL) | 0.76 (0.5,1.2) | 0.7 (0.4,1.0) | 0.121 |
| diuretics, n (%) | 432 (86.9) | 184 (92) | 0.058 |
| Use of ACEIs/ARBs, n (%) | 66 (13.3) | 28 (14) | 0.801 |
| angiography, n (%) | 271 (54.5) | 107 (53.5) | 0.806 |
| MCV (fL) | 90.6 (88.1,93.5) | 90.5 (88.125,93.2) | 0.393 |
| Type of surgery, n (%) |  |  | 0.762 |
| MV | 70 (14.1) | 24 (12) |  |
| AV | 96 (19.3) | 45 (22.5) |  |
| MV+TV | 249 (50.1) | 102 (51) |  |
| MV+AV | 21 (4.2) | 9 (4.5) |  |
| MV+TV+AV | 61 (12.3) | 20 (10) |  |
| bleeding volum>700mL, n (%) | 265 (53.3) | 103 (51.5) | 0.663 |
| urine volume>1200mL, n (%) | 233 (47) | 109 (54.5) | 0.072 |
| CPB time (min) | 123 (100,153) | 119 (98.75,145.25) | 0.2 |
| ACx time (min) | 87 (68,113) | 86 (70,113) | 0.854 |
| repeat CPB, n (%) | 19 (3.8) | 6 (3) | 0.597 |
| RBC transfusion, n (%) | 203 (40.8) | 57 (28.5) | 0.002 |
| plasma transfusion, n (%) | 155 (31.2) | 38 (19) | 0.001 |
| nadir hematocrit (L/L) | 39 (37,43) | 40 (37,43) | 0.148 |
| VIS (score) | 5 (5,7) | 5 (5,7) | 0.050 |
| fluid balance (mL) | 230 (-350,721) | 42.5 (-367.75,588) | 0.477 |

**Abbreviations:** BMI: body mass index; CHD: coronary heart disease; AF: atrial fibrillation; LVEF: left ventricular ejection fraction; Hb: hemoglobin; WBC: white blood cells; PLT: platelets; Alb: albumin; Scr: serum creatinine; eGFR: estimated glomerular filtration rate; PT: prothrombin time; APTT: activated partial thromboplastin time; FDP: fibrinogen Degradation Products; ACEIs: angiotensin-converting enzyme inhibitors; ARBs: angiotensin receptor blockers; MCV: mean corpuscular volume; MV: mitral valve; AV: aortic valve; TV: tricuspid valve; RBC: red blood cell; CPB: cardiopulmonary bypass; ACx: aortic cross-clamping; HCT: hematocrit; VIS: vasoactive–inotropic score.
